# Influence of cervical preflaring on apical transportation in curved root canals instrumented by reciprocating file systems

**DOI:** 10.1186/s12903-015-0137-0

**Published:** 2015-11-23

**Authors:** Neisiana Barbieri, Denise Piotto Leonardi, Marina Samara Baechtold, Gisele Maria Correr, Marilisa Carneiro Leão Gabardo, João César Zielak, Flares Baratto-Filho

**Affiliations:** Department of Dentistry, Positivo University, Curitiba, Rua Professor Pedro Viriato Parigot de Souza, 5300 Campo Comprido, 81280-330 Curitiba, PR Brazil

**Keywords:** Apical transportation, Reciprocating motion, Root canal preparation, WaveOne

## Abstract

**Background:**

The aim of this study was to evaluate the influence of cervical preflaring on apical transportation in curved root canals prepared using the Reciproc and WaveOne reciprocating file systems.

**Methods:**

Sixty curved canals were instrumented using Reciproc R25 and WaveOne Primary files, with and without preflaring (*n* = 15). A double-digital technique was used to digitally superimpose the file before instrumentation (#15 K-file) on the final apical reciprocating file (#25/08). The angle between the tip of the initial and final file was measured and recorded. Groups were compared using the Kruskal–Wallis test, and significance was set at *p* < 0.05.

**Results:**

The mean and standard deviation for apical transportation was 0.93 ± 2.48 for the Reciproc Group, 0.84 ± 1.94 for the Preflaring + Reciproc Group, 0.40 ± 1.14 for the WaveOne Group, and 0.83 ± 2.20 for the Preflaring + WaveOne Group. No statistically significant differences were found among the groups (*p* = 0.9509).

**Conclusions:**

Under the conditions of this study, cervical preflaring did not influence apical transportation in curved root canals instrumented using Reciproc R25 and the WaveOne Primary files. Based on the in vitro measurements of apical transportation, the reciprocating files may be used without preflaring in curved root canals.

## Background

To reduce infection and to prevent or treat apical periodontitis, it is important that the root canal is mechanically shaped using a technique that cleans and preserves its original anatomy without deviations such as ledging and apical transportation [1, 2]. Errors in this procedure hamper efficient cleaning of the root canals, leading to the retention of infected debris and residual microorganisms. Incorrect mechanical shaping can also result in decreased fracture resistance of the root, which may compromise the treatment outcome [3].

Apical transportation is often caused by mechanical instrumentation with large files that are more effective in removing infected tooth structure than small files [4, 5]. Enlarging the root canal to a larger diameter removes more debris and promotes better cleaning of the apical third, while allowing maximal contact of the irrigant with the apical debris [6, 7]. Therefore, mechanical preparation of curved root canals remains challenging [8].

Nickel titanium (NiTi) rotary instruments reduce working time, operator fatigue, and procedural errors during preparation [9]. Owing to their specific design and flexibility, NiTi rotary files make root canal preparation safer and more efficient because they provide better centering in the canal when compared with stainless-steel hand files even in severely curved root canals [10, 11]. However, NiTi rotary files are susceptible to fracture, especially when used in narrow and curved root canals [12].

A recently developed concept of root canal preparation aims to reduce working time and the incidence of fracture by using a single file under a reciprocating motion [13, 14]. Different reciprocating systems have different design features such as taper, depth of flutes, cross-sectional design, and number of spirals per unit length. The ability to shape curved root canals without causing apical transportation may be affected by these systems’ characteristics [15].

Studies show that the anatomy of the root canal may be modified when shaped by reciprocating systems. Thus, additional technical clinical procedures may be needed [16, 17]. It is known that cervical preflaring allows a more straight-line access into the root canal, improving the apical anatomical diameter determination [18, 19]. Moreover, apical transportation may be reduced in root canals that have been preflared [20].

Considering these findings, the present study aimed to evaluate the influence of cervical preflaring on apical transportation in curved root canals prepared using Reciproc R25 and WaveOne Primary file systems.

## Methods

This research has been conducted in full accordance with the World Medical Association Declaration of Helsinki and was approved by the Local Ethics Committee of the Positivo University, Brazil (43104/2012). Teeth used in this study were extracted by periodontal reasons. All pacients have consented the treatment by writing. Sixty mesiobuccal root canals of maxillary molars with complete root formation were selected. Only mesiobuccal (MB) root canals that allowed the placement of a #10 K-Flexofile (Dentsply Maillefer, Ballaigues, Switzerland) to the apical foramen were used. Access cavities were prepared with round diamond and Endo-Z burs (Dentsply). The working length (WL) of each canal was established by inserting a #10 K-Flexofile into the root canal until the file tip became visible through the apical foramen under a stereomicroscope at 20× magnification, then subtracting 1 mm.

An apparatus was manufactured in acrylic resin to provide a fixed position for the digital dental X-ray sensor and cone alignment. A #15 K-Flexofile was inserted into the root canal at the WL and a digital radiograph (Kodak RVG 6100; Kodak, Rochester, NY, USA) was obtained to register the initial apical curvature. The root canal curvature angle and radius were measured according to the method of Pruett et al. [21] using the AutoCAD 2008 program (Autodesk Inc, San Rafael, CA, USA). The samples were divided into four groups (*n* = 15) with similar canal lengths, angles, and radii of curvature. The angles of curvature ranged from 20° to 40° and the radius 6 mm.

A double-digital radiographic technique was used to analyze apical transportation [15]. Two preinstrumentation digital radiographs with buccolingual and mesiodistal views were obtained of each tooth with a #15 K-Flexofile at the WL.

Group 1 was assigned to preparation with Reciproc #25.08 (VDW GmbH, Munich, Germany) at the WL. Group 2 was assigned to cervical preflaring with SX (Dentsply Maillefer) and preparation with Reciproc #25.08 at the WL. Group 3 was assigned to preparation with WaveOne #25.08 (Dentsply Maillefer) at the WL. Group 4 was assigned to cervical preflaring with SX (Dentsply Maillefer) and preparation with WaveOne #25.08 (Dentsply Maillefer) at the WL.

The instruments were activated with a 6:1 reduction hand piece powered by a torque-limited electric motor (X-Smart Plus, Dentsply Maillefer) using the preset program Reciproc for the Reciproc instruments and the preset program WaveOne for the WaveOne instruments. SX instruments were used with the same electric motor using the preset program for the SX instrument. A glide path up to #15 Flexofile was set before instrumentation. The root canals were irrigated with 5.25 % NaOCl delivered with a syringe and a 30-gauge side-vented irrigating tip.

Reciprocating files were gradually inserted into the root canal with cycles of three pecks, removed and cleaned, and the root canal was irrigated. Each instrument was used in four root canals. After preflaring, reciprocating files WL was repositioned in 1 mm from the root apex when it was observed working length changes. When preparation was complete, the #25.08 Reciproc or WaveOne file was placed into the root canal at the WL, the tooth was repositioned on the radiographic apparatus, and two post-instrumentation radiographs were taken.

**Fig. 1:**
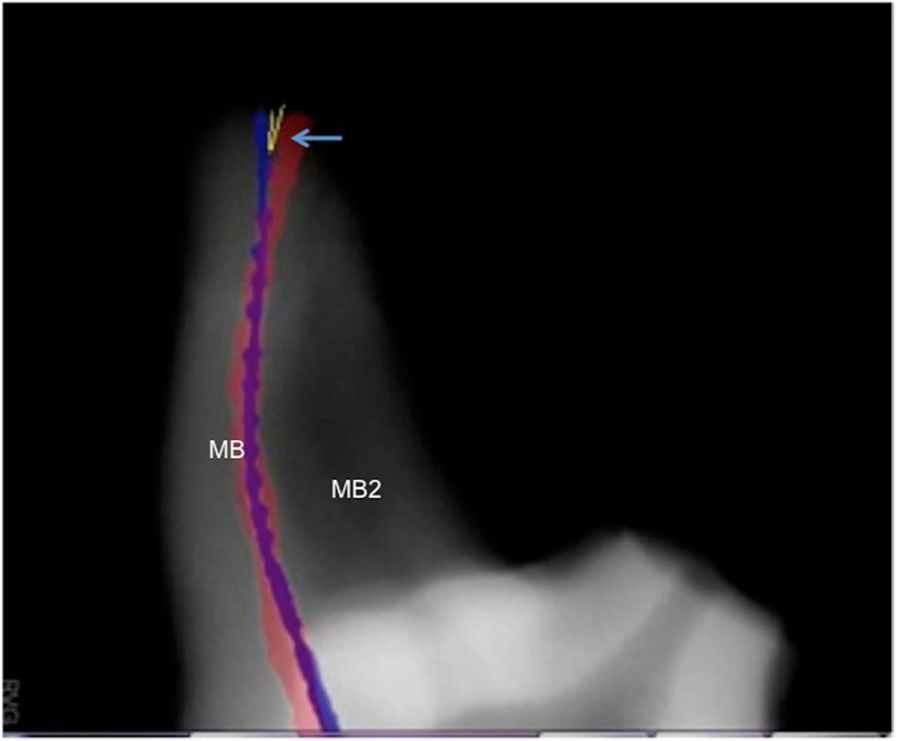
Angle between the tip of the initial and final file (arrow)

Adobe Photoshop software (Adobe Systems Inc., San Jose, CA, USA) was used to superimpose each post-instrumentation image onto its matching pre-instrumentation image. The images were imported into the freely available ImageJ software (http://imagej.nih.gov/ij/) to analyze the apical transportation. The angle between the tip of the initial and final file was measured and the higher value (buccolingual or mesiodistal) was recorded (Fig. 1).

Data were evaluated for normal distribution using the Kolmogorov–Smirnov and Lilliefors test. Homogeneity was analyzed using the Levene test. Since the data did not follow a normal distribuition the groups were compared using the non-parametric Kruskal-Wallis test. Significance was set at *p* < 0.05 (Statistica 7.0, Statsoft, 2004).

## Results

The mean and standard deviation of the angle between the tip of the initial and final files were 0.93° ± 2.48° in the Reciproc Group, 0.84° ± 1.94° in the Preflaring + Reciproc Group, 0.40° ± 1.14° in the WaveOne Group, and 0.83° ± 2.20° in the Preflaring + WaveOne Group. The Kruskal–Wallis test showed no statistically significant differences among the groups (*p* = 0.9509).

## Discussion

The single-file principle for root canal preparation has advantages of ease of learning and cost effectiveness [22]. Yared [23] introduced this technique using the ProTaper F2 instrument in a reciprocating motion.

Two further root canal preparation systems were introduced featuring reciprocating motion: Reciproc (VDW) and WaveOne (Dentsply Maillefer). These instruments are made of M-wire NiTi alloy produced by an innovative thermal treatment process [24]. Both instruments were designed to promote improved shaping, lower the rate of instrument failure, and reduce the number of procedural steps. Recently, there is a new generation of reciprocating file, the WaveOne GOLD whose design has also been optimized to increase cutting efficiency.

Apical transportation may reduce root canal disinfection and difficult sealing ability of the root filling [25]. Studies have assessed apical transportation in curved root canals prepared with reciprocating instruments [26–29]. However, the effect of cervical preflaring when using Reciproc and WaveOne instruments has not been assessed.

In our study, apical transportation was minimal in all groups even when cervical preflaring was not performed. This may be due to the initial width of the root canal in which a #10 K-file was easily inserted. Maybe if the initial root canal width was smaller the preflaring procedure could have shown effects on the apical transportation. There were no differences between WaveOne and Reciproc groups with no preflaring. Previous studies obtained similar results when using reciprocating instruments in curved canals assessed by similar radiographic techniques [17, 30]. Furthermore, no differences between the two reciprocating systems have been observed in studies using different techniques [16, 31]. Radiographic superimposition techniques are frequently used to assess apical transportation. Despite the inability to achieve a volumetric analysis, the radiographic method is inexpensive, easy to perform, and reliable [32]. In this present study, each sample was positioned on the radiographic apparatus in order to achieve standardized radiographic images and to reduce errors.

The results from our study may be related to the characteristics of the instruments. The Reciproc R25 and the WaveOne Primary have a size 25 non-cutting tip with a taper of 0.08. Non-cutting tips have been shown to produce apical transportation in severely curved canals [33]. Thus, taper size plays an important role in apical transportation: the larger the taper of the instrument, the lower is its flexibility [34]. However, in root canals with apical curvature ranging from 20° to 40°, these instruments may be able to retain their original conformation. Similar results were observed in a study by Capar et al. [16], who assessed apical transportation in curvatures ranging from 20° to 40° using R25 and WaveOne Primary files.

Besides, the results may be also related to the instrument movement. Reciprocation motion occurs in the direction of the flutes, which are ground angled to the left, being the opposite of balanced force movement kinematics [35, 36]. As previously stated, the reciprocating movement aims to minimize the risk of instrument fracture caused by torsional stress once the angle of the counterclockwise rotation is designed to be smaller than the elastic limit of the instrument [22]. A smaller angle in a clockwise direction allows the file to be immediately disengaged and safely progress along the canal path, while reducing the screwing effect. These conditions raise questions about the influence of this movement on apical transportation. Giuliani et al. [37] observed when preparing S-shaped canals that full-sequence ProTaper Universal NiTi files used in a reciprocating motion exhibited better shaping effects than full-sequence ProTaper Universal NiTi files used in a conventional motion.

The results of any in vitro study must be carefully applied to the clinical practice. From our data, it can be conclude that cervical preflaring did not influence the occurrence of apical transportation when curved root canals were prepared with Reciproc R25 and WaveOne Primary files. Both Reciproc R25 and WaveOne Primary file systems performed similarly in curved root canals.

## Abbreviations

NiTi: Nickel titanium
WL: Working length
